# Contemporary series of transsphenoidal microsurgery in pediatric patients

**DOI:** 10.1007/s10143-025-04019-6

**Published:** 2026-01-23

**Authors:** Stefanie Ott, Nora Ramdani, Lasse Dührsen, Franz L. Ricklefs, Roman Rotermund, Jörg Flitsch, Alice Ryba

**Affiliations:** 1 Department of Neurosurgery, Medical Center Hamburg-Eppendorf, Hamburg, Germany; 2 Department of Neuroradiology, Medical Center Hamburg-Eppendorf, Hamburg, Germany; 3 Department of Neurosurgery, Diako Hospital, Flensburg, Germany

**Keywords:** Pediatric, Pituitary, Craniopharyngioma, Adenoma

## Abstract

Pituitary tumors are rare in the pediatric population, accounting for less than 10% of childhood tumors. Craniopharyngiomas are the most common pediatric sellar lesions, but pituitary adenomas/PitNETs also occur, representing 2–8% of all sellar lesions. This study explores a decade of transsphenoidal microsurgery on pediatric patients, examining perioperative features including complications, clinical outcomes and anatomical peculiarities. This retrospective study included a total of 147 transsphenoidal surgeries performed by a single surgeon on 122 pediatric patients under the age of 18 between 2013 and 2023. Parameters assessed included age, gender, clinical hormone production, histopathological and radiographic parameters, surgical features, postoperative complications and recurrences rate. Among 122 pediatric patients with pituitary lesions, we found that craniopharyngiomas were the most common entity (34.4%), especially in children under 10 years old (58.6%). Pituitary adenomas/PitNETs accounted for 31.1% and were more prevalent in older children, with corticotrophic and lactotrophic adenomas being the most common subtypes. Younger patients needed significantly more time for surgery, with the longest durations observed in the 3–9 years age group and the shortest in the 16–17 years age group. Additionally, sphenoid sinus pneumatization increased with age and was inversely correlated with the duration of surgery. The current study is one of the largest to date on pediatric pituitary region lesions shedding light on demographic, clinical and histopathological features. The age-dependent distribution pattern highlights the prominence of craniopharyngiomas in younger patients, while PPAs became increasingly prevalent in those older than 10 years.

**Supplementary Information:**

The online version contains supplementary material available at 10.1007/s10143-025-04019-6.

## Introduction

Sellar lesions are rare in the pediatric population, accounting for less than 10% of childhood tumors [1]. These tumors can arise from various entities, including adenomas, craniopharyngiomas, Rathke’s cleft cysts, germ cell tumors, and others [2]. Most studies have focused on craniopharyngiomas since they constitute the majority of pediatric sellar lesions. However, pediatric pituitary adenomas (PPAs), representing only 2–8% of sellar lesions [2], also occur and can be further categorized into secreting and non-secreting types. Unlike in the adult population, non-functional PPAs are even rarer in children (5–10% of PPAs), and the incidence of secreting types varies across different age groups [1, 3, 4]. Additionally, a variety of other lesions can arise in the sellar region, and not all require surgical intervention. Clinical presentations range from nonspecific symptoms to more common visual disturbances and pituitary dysfunction [1], which may manifest as growth arrest, delayed puberty, or symptoms attributed to specific secreting adenomas. Given the significant impact of endocrinological impairments on a child’s development, careful preoperative and postoperative management is crucial when treating pediatric patients with sellar lesions.

In recent years, only a few series on the surgical management of pediatric sellar lesions have been published, some focusing on the endoscopic endonasal technique and its safety concerning the maturation of the pediatric skull base and facial growth [5]. To date, most reviews include a limited number of pediatric patients [1, 6, 7], and only a restricted number of retrospective studies have evaluated larger pediatric populations through multicenter reviews or databases [3, 4]. Few studies have compared different surgical transsphenoidal techniques, namely the microsurgical and endoscopic approaches, both showing satisfying results in the current literature [7]. In children, special considerations for transsphenoidal surgery include a smaller midface as the nasal corridor for approaching the sellar region, a narrow intercarotid distance, and age-dependent variations in sphenoid sinus pneumatization [8, 9]. Sphenoid bone pneumatization begins around 10 months of age, accelerates during childhood between ages 3 and 6, and continues until the late third decade of life. Therefore, special attention and preoperative planning are necessary to address these anatomical changes during pediatric sellar surgery [8].

Here, we report a single-center experience spanning a decade of transsphenoidal microsurgical treatment in pediatric patients. This study aimed to evaluate age-dependent anatomical and clinical features influencing the feasibility and safety of transsphenoidal microsurgery in pediatric sellar lesions.

Secondary objectives were to describe entity-specific perioperative outcomes and identify potential risk factors for postoperative complications or recurrence.

## Materials and methods

In this retrospective single-center analysis out of a total of 3113 transsphenoidal surgeries, *n* = 147 transsphenoidal surgeries (microscopic or 3D-videomicroscopic) were performed by one surgeon (JF) on pediatric patients (< 18yrs) between January 2013 and December 2023 at our institution. Herein *n* = 25 patients had a repeated surgery at our institution. A range of clinical, radiological, surgical and histopathological parameters as well as surgical outcome were extracted from hospital records and retrospectively analyzed.

Written consent was obtained from their legal guardians and anonymized data was analyzed in accordance with the local and ethical guidelines (2023–300418-WF) and the 1964 declaration of Helsinki.

### Patients’ characteristics

Patients characteristics encompassed age, gender, tumor entity, preoperative symptoms, surgical features postoperative complications and outcome at time of follow-up. Herein, postoperative complications included CSF leakage, arginine vasopressin disorder (Diabetes insipidus), SIADH, epistaxis, visual impairment, meningitis, and new pituitary insufficiency. The diagnosis of hypopituitarism, SIADH and DI was based on clinical assessment in conjunction with serial postoperative laboratory measurements. Regular blood sampling was performed in all patients during the early postoperative course, including daily serum electrolyte panels and hormonal profiles (ACTH, cortisol, TSH, free T4, LH/FSH, testosterone/estradiol, and IGF-1), as clinically indicated. Abnormal laboratory findings in combination with relevant clinical signs such as hypotension, hyponatremia, polyuria, or changes in mental status prompted endocrinologic evaluation and treatment. Tumors were classified according to their histopathological report. In all cases, the chosen surgical approach was an endonasal transsphenoidal technique, performed by an experienced pituitary surgeon (JF). Surgical duration was measured in minutes, starting from the initial incision to the final closure. The extent of resection (EOR) for pituitary adenomas was determined using a combined approach, incorporating the surgeon’s intraoperative assessment and operative reports, as well as postoperative follow-up imaging obtained within 3 to 6 months after surgery. Immediate postoperative MRI was not routinely performed. Follow-up was conducted via the outpatient department and/or written correspondence on pituitary functional assessment and follow up imaging with MRI. Remission of functioning pituitary adenomas was defined as normalization of the respective hormone levels and absence of residual or recurrent tumor on follow-up contrast-enhanced MRI. To obtain balanced groups and robust estimates, we defined age strata by the quartiles of the study population, yielding the bands 3–9, 10–12, 13–15, and 16–17 years. These quartiles coincide with known phases of sinonasal and skull-base development, including the acceleration of sphenoid sinus pneumatization in early–mid childhood and consolidation during adolescence. To mitigate residual concerns about categorization, age was additionally modeled as a continuous variable in correlation analyses, which yielded results consistent with the grouped comparisons.

### Radiographic parameters

Tumor volumetrics were measured with the approximation formula length*width*height/2 on T1-weighted MRI with contrast enhancement. Furthermore, in pituitary adenomas/PitNETs preoperative tumor invasiveness was assessed using Knosp grading.

**Fig. 1:**
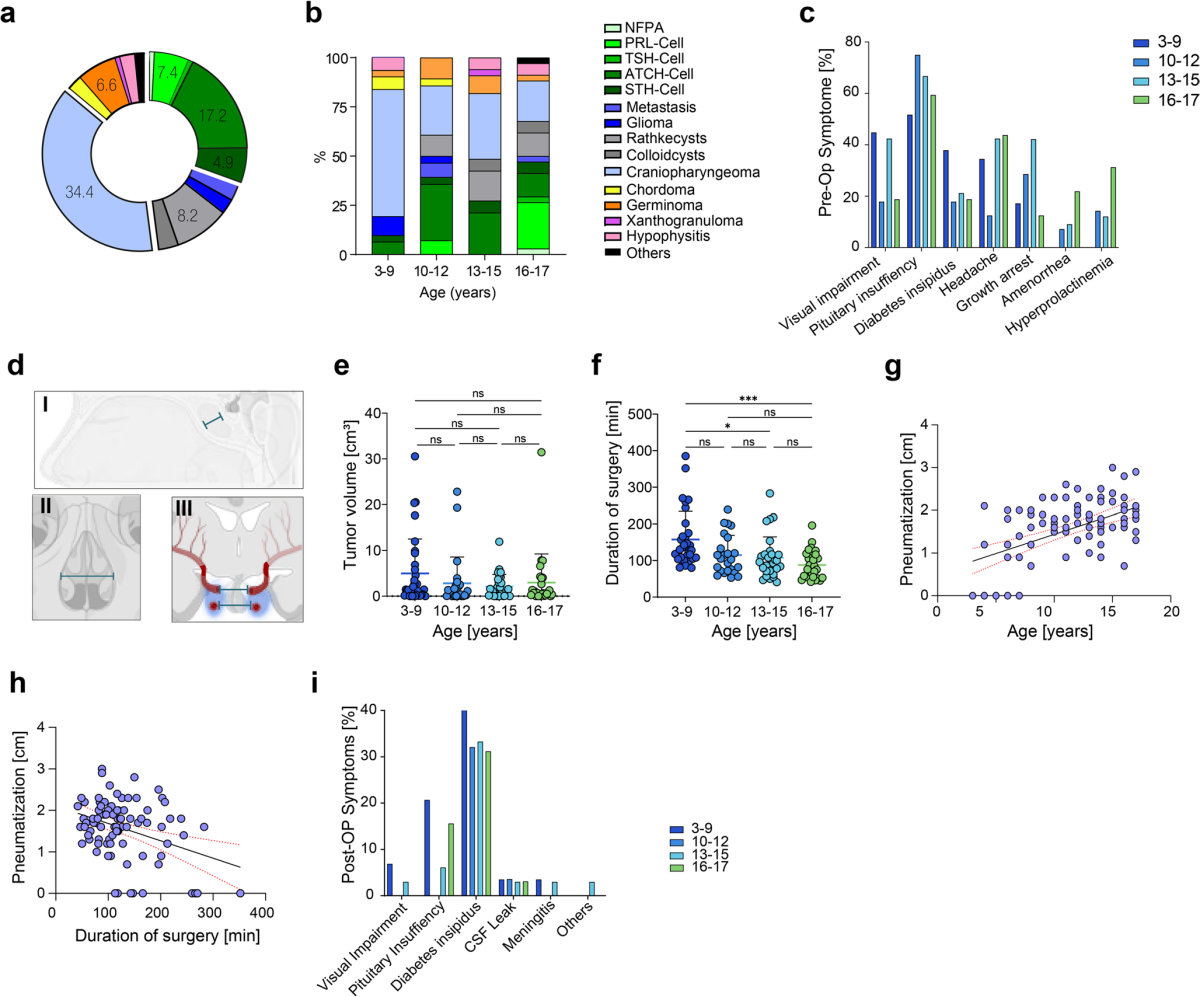
Age dependency in transsphenoidal sellar surgery - **a** The distribution of lesion entities in the sellar region among all pediatric patient with craniopharyngeomas as the most common entitiy (34.4%), followed by pituitary adenoma (31.5%). **b** Age-dependent distribution of sellar lesions categorized into four groups (ages 3–9, 10–12, 13–15, and 16–17 years). **c** Age-related preoperative symptoms. **d** Illustration of radiographic measurements of *I*: pneumatization of the sphenoid sinus, *II*: apertura piriformis, *III*: Upper and lower intercarotid distance. **e** Tumor volume of sellar lesions categorized by age **f** Surgical duration varied across age groups, notably showing significantly longer surgeries for patients aged 3–9 years compared to those aged 13–15 years (157.3 vs. 107.6 min., *p* = 0.0358) and 16–17 years (157.3 vs. 88.0 min., *p* = 0.0004). **g** The extent of pneumatization correlated positively with patient age (R²=0.2721). **h** The extent of pneumatization of the sphenoid sinus correlated negatively with duration of surgery (R²=0.1311). **i** Postoperative complications in pediatric pituitary lesions: The age group of 3–9 years exhibited the highest frequency of postoperative complications (93.1%, *n* = 27/29)

A neuroradiologist retrospectively assessed anatomical variations in children that could impact the transsphenoidal approach, including sphenoid sinus pneumatization, piriform aperture size, and intercarotid distance on preoperative MR-images. The degree of pneumatization was evaluated using sagittal T1-weighted MRI scans to estimate the amount of drilling needed to reach the sellar region. The size of the piriform aperture was determined using coronal (T1-weighted) MRI scans, confirming an adequately wide nasal access point. Intercarotid distance within the sellar region was assessed using axial T1-weighted MRI scans (Fig. 1d).

### Surgical parameters

All patients underwent transsphenoidal surgery performed by a specialized pituitary surgeon at our institution. From 2013 to 2018, the surgeries were conducted using a microscope, whereas after 2018, an exoscope was used for all procedures. Patients were positioned according to the modified Hardy-Lüdecke technique, in a semi-sitting position facing the surgeon. The surgery was performed using a mono-nostril approach, with the sphenoid sinus accessed via a transseptal preparation of the posterior part of the lamina perpendicularis. Small Kocher specula were installed to allow for bimanual preparation, and a sagittal x-ray was used to confirm the working corridor and direction. Drilling may be required to reach the sella floor, depending on pneumatization of the sphenoid sinus. For enhanced visualization of microsurgical details, micro-mirrors were employed for the lateral aspects of lesionectomy. In cases of CSF fistula, a muscle patch, fibrin glue and optional Tachosil were used to achieve a watertight closure.

### Statistical analysis

Statistical analyses were carried out on GraphPad Prism 9.0 software. Figures were generated using adobe illustrator (Version 29.8.1). Continuous variables were analyzed using unpaired two-tailed independent Student’s t-test for comparison of two groups, whereas ANOVA is used to compare the means of continuous scaled data of three groups or more. Chi-square was utilized for comparison of categorial variables within two-groups or Fisher’s exact test for expected frequencies below 5. Correlations between continuous variables were assessed using Pearson’s correlation coefficient and corresponding coefficient of determination (R^2^). Statistical significance was defined as *p* < 0.05. For key comparisons, 95% confidence intervals (CI) were calculated and are reported in the results section. The primary outcome of this study was to evaluate age-dependent differences in anatomical and surgical parameters, particularly the relationship between patient age, sphenoid sinus pneumatization, and operative time. Secondary outcomes included the EOR, postoperative complications, and recurrence rates stratified by pathology (craniopharyngioma vs. PitNET).

## Results

Out of a total of *n* = 3113 transsphenoidal surgeries performed at the University Medical Center Hamburg-Eppendorf between the years 2013 and 2023, we analyzed *n* = 122 individual pediatric patients that underwent transsphenoidal surgery, of whom *n* = 25 underwent a repeated transsphenoidal surgery at our hospital. The main characteristics of primarily operated pediatric patients and their age-related differences are summarized in Table 1.

**Table 1:** Characteristics of the whole pediatric cohort and age-related differences

	Total	3–9 years	10–12 years	13–15 years	16–17 years	*p* value
Total						
Patients, n (%)	122 (100)	29/122 (25.4)	28/122 (23)	33/122 (27)	32/122 (26.2)	
Clinial Data						
Median Age [yrs] (Range)	13 (3–17)	6 (3–9)	11 (10–12)	14 (13–15)	16 (16–17)	
Gender - n (%)						0.3023
Female	62 (50.8)	17/29 (58.6)	10/28 (35.7)	17/33 (51.5)	18/32 (56.3)	
Male	60 (49.2)	12/29 (41.4)	18/28 (64.3)	16/33 (48.5)	14/32 (43.7)	
Preoperative						
Visual Impairment - n (%)	38 (31.1)	13/29 (44.8)	5/28 (17.9)	14/33 (42.4)	6/32 (18.8)	
Pituitary Insufficiency - n (%)	77 (63.1)	15/29 (51.7)	21/28 (75)	22/33 (66.7)	19/32 (59.4)	
Diabetes insipidus - n (%)	30 (24.6)	12/29 (41.4)	5/28 (17.9)	7/33 (21.2)	6/32 (18.8)	
SIADH - n (%)	0 (0)	0/29 (0)	0/28 (0)	0/33 (0)	0/32 (0)	
Hyperprolactinemia - n (%)	18 (14.8)	0/29 (0)	4/28 (14.3)	4/33 (12.1)	10/32 (31.3)	
Headache - n (%)	44 (36.1)	10/29 (34.5)	6/28 (12.5)	14/33 (42.4)	14/32 (43.8)	
Growth arrest - n (%)	31 (25.4)	5/29 (17.2)	8/28 (28.6)	14/33 (42.4)	4/32 (12.5)	
Amenorrhoe - n (%)						
Primary	4 (3.3)	0/29 (0)	1/28 (3.6)	1/33 (3)	2/32 (6.3)	
Secondary	4 (3.3)	0/29 (0)	0/28 (0)	0/33 (0)	4/32 (12.5)	
NA	1 (0.8)	0/29 (0)	0/28 (0)	1/33 (3)	0/32 (0)	
Pubertas tarda - n (%)	2 (1.6)	0/29 (0)	0/28 (0)	1/33 (3)	1/32 (3.1)	
Radiographic Data						
Tumor volume [cm³] - Mean *±* SD	3.19 *±* 5.76	4.9 *±* 7.5	2.8 *±* 5.8	2.1 *±* 2.6	2.9 *±* 6.3	0.4191
Upper Intercarotid distance [cm] - Mean *±* SD	1.5 *±* 0.4	1.9 *±* 0.3	1.9 *±* 0.4	2.1 *±* 0.4	2.0 *±* 0.3	0.4688
Lower Intercarotid distance [cm] - Mean *±* SD	2.0 *±* 0.4	1.9 *±* 0.3	1.9 *±* 0.5	2.1 *±* 0.4	2.0 *±* 0.3	0.0621
Apertura piriformis [cm] - Mean *±* SD	1.6 *±* 0.3	1.5 *±* 0.3	1.6 *±* 0.3	1.6 *±* 0.2	1.6 *±* 0.3	0.0938
Pneumatization sinus sphenoidalis [cm] - Mean *±* SD	1.6 *±* 0.7	1.0 *±* 0.8	1.8 *±* 0.4	1.8 *±* 0.5	1.7 *±* 0.5	**< 0.0001**
Surgical Data						
Duration of surgery [min] - Mean *±* SD	114.8 *±* 58.5	157.3 *±* 76.7	115.1 *±* 53.8	107.6 *±* 57.4	88.0 *±* 37.9	**0.0009**
EOR - (%)						0.6899
Total	47 (38.5)	11/29 (37.9)	9/28 (32.1)	14/33 (42.4)	13/32 (40.6)	
Subtotal	53 (43.4)	12/29 (41.4)	14/28 (50)	12/33 (36.3)	15/32 (35.7)	
Biopsy	18 (14.8)	6/29 (20.7)	3/28 (10.7)	2/33 (6.1)	3/32 (9.4)	
NA	4 (3.3)	0/29 (0)	2/28 (7.2)	5/33 (15.5)	1/32 (3.1)	
Histology - n (%)						
Non-secreting Adenoma	1 (0.8)	0/29 (0)	0/28 (0)	0/33 (0)	1/32 (3.1)	
Prolactinoma	9 (7.4)	0/29 (0)	2/28 (7.2)	0/33 (0)	7/32 (21.9)	
TSHnom	1 (0.8)	0/29 (0)	0/28 (0)	0/33 (0)	1/32 (3.1)	
ATCH-Cell	21 (17.2)	2/29 (6.9)	8/28 (28.6)	7/33 (21.2)	4/32 (12.5)	
Acromegalia (STH)	6 (4.9)	1/29 (3.5)	1/28 (3.6)	2/33 (6.1)	2/32 (6.3)	
Metastasis	3 (2.5)	0/29 (0)	2/28 (7.2)	0/33 (0)	1/32 (3.1)	
Glioma	4 (3.8)	3/29 (10.3)	1/28 (3.6)	0/33 (0)	0/32 (0)	
Rathkecysts	10 (8.2)	0/29 (0)	3/28 (10.7)	4/33 (12.1)	3/32 (9.4)	
Colloidcysts	4 (3.3)	0/29 (0)	0/28 (0)	2/33 (6.1)	2/32 (6.3)	
Craniopharyngeoma	42 (34.4)	17/31 (58.6)	7/28 (25)	11/33 (33.3)	7/32 (21.9)	
Chordoma	3 (2.5)	2/29 (6.9)	1/28 (3.6)	0/33 (0)	0/32 (0)	
Germinoma	8 (6.6)	1/29 (3.5)	3/28 (10.7)	3/33 (9.1)	1/32 (3.1)	
Xanthogranuloma	2 (1.6)	0/29 (0)	0/28 (0)	2/33 (6.1)	0/32 (0)	
Hypophysitis	6 (4.9)	3/29 (10.3)	0/28 (0)	1/33 (3)	2/32 (6.3)	
Encephalocele	1 (0.8)	0/29 (0)	0/28 (0)	1/33 (3)	0/32 (0)	
Outcome						
Postoperative Deficits	65 (53.3)	27/29 (93.1)	8/28 (28.6)	16/33 (48.5)	14/32 (43.7)	**< 0.0001**
Transient						
Diabetes insipidus - n (%)	12 (9.8)	3/29 (10.3)	4/28 (14.3)	2/33 (6.1)	3/32 (9.4)	0.6619
Persistent after surgery	0 (0)	0/29 (0)	0/28 (0)	0/33 (0)	0/32 (0)	
New onset	12 (9.8)	3/29 (10.3)	4/28 (14.3)	2/33 (6.1)	3/32 (9.4)	
Visual Impairment - n (%)	0 (0)	0/29 (0)	0/28 (0)	0/33 (0)	0/32 (0)	
SIADH - n (%)	0 (0)	0/29 (0)	0/28 (0)	0/33 (0)	0/32 (0)	
Epistaxis - n (%)	0 (0)	0/29 (0)	0/28 (0)	0/33 (0)	0/32 (0)	
CSF Leak - n (%)	4 (3.3)	1/29 (3.5)	1/28 (3.6)	1/33 (3.0)	1/32 (3.1)	
Meningitis - n (%)	2 (3.5)	1/29 (3.5)	0/28 (0)	1/33 (3)	0/32 (0)	
Others - n (%)	1 (0.8)	0/29 (0)	0/28 (0)	1/33 (3)	0/32 (0)	
Permanent						
Visual Impairment - n (%)	3 (2.5)	2/29 (6.9)	0/28 (0)	1/33 (3)	0/32 (0)	
New Pituitary Insufficiency - n (%)	13 (10.7)	6/29 (20.7)	0/28 (0)	2/33 (6.1)	5/32 (15.6)	
Diabetes insipidus - n (%)	30 (24.6)	14/29 (48.3)	3/28 (10.7)	8/33 (24.2)	5/32 (15.6)	**0.0045**
Persistent after surgery	22 (18)	11/29 (37.9)	2/28 (7.2)	5/33 (15.1)	4/32 (12.5)	
New onset	8 (6.6)	3/29 (10.3)	1/28 (3.6)	3/33 (9.1)	1/32 (3.1)	
SIADH - n (%)	0 (0)	0/29 (0)	0/28 (0)	0/33 (0)	0/32 (0)	
Epistaxis - n (%)	0 (0)	0/29 (0)	0/28 (0)	0/33 (0)	0/32 (0)	
CSF Leak - n (%)	0 (0)	0/29 (0)	0/28 (0)	0/33 (0)	0/32 (0)	
Meningitis - n (%)	0 (0)	0/29 (0)	0/28 (0)	0/33 (0)	0/32 (0)	
Others - n (%)	0 (0)	0/29 (0)	0/28 (0)	0/33 (0)	0/32 (0)	
Remission						
Recurrence - n (%)	19 (15.6)	8/29 (27.6)	2/28 (7.2)	3/33 (9.1)	6/32 (18.8)	0.1118
Tumor rest - n (%)	45 (36.8)	16/29 (55.2)	6/28 (12.5)	14/33 (42.4)	9/32 (28.1)	**0.0363**
Adjuvant therapy - n (%)	32 (26.2)	12/29 (41.4)	5/28 (17.9)	6/33 (18.2)	9/32 (27.1)	0.1317
Re-Surgery	16 (13.1)	8/29 (27.6)	2/28 (7.2)	1/33 (3)	5/32 (15.6)	
Combined w. CTx	1 (0.8)	1/29 (3.5)	0/28 (0)	0/33 (0)	0/32 (0)	
Combined w. RTx	3 (2.5)	2/29 (6.9)	1/28 (3.6)	0/33 (0)	0/32 (0)	
RTx	9 (7.4)	1/29 (3.5)	1/28 (3.6)	5/33 (15.5)	2/32 (6.3)	
CTx	1 (0.8)	0/29 (0)	0/28 (0)	0/33 (0)	1/32 (3.1)	
SSA	2 (1.6)	0/29 (0)	1/28 (3.6)	0/33 (0)	1/32 (3.1)	

The median age was 13 years (range: 3–17). Overall, both genders were nearly equally represented, with 50.8% female and 49.2% male patients and no significant gender differences were observed across age groups (*p* = 0.3023). In general, craniopharyngioma was the most common histological entity (*n* = 42, 34.4%) of all surgically addressed pediatric patients and predominantly represented in the 3–9 years group (58.6%, *n* = 17) (Fig. 1a). The distribution of pathologies varied among age groups, with significant representation in certain age categories (Fig. 1a). In contrast, PPA/PitNETs were most common in the 10–12 years age group (39.4%, *n* = 11), with ACTH-secreting adenomas being the most frequent subtype (28.6%, *n* = 8) (Fig. 1b).

Preoperative visual impairment was present in 31.1% of patients (*n* = 38), with the highest incidence in the 3–9 years age group (44.8%, *n* = 13) (Fig. 1c). Pituitary insufficiency was the most common preoperative condition, affecting 63.1% of patients (*n* = 77), particularly prominent in the 10–12 years group (75%, *n* = 21). DI was noted in 23.8% of patients, and hyperprolactinemia in 14.8%, with a notable increase in the 16–17 years group (31.3%, *n* = 10). Headaches were reported by 36.1% of patients (*n* = 44), with the highest frequency in the 13–15 years (42.4%, *n* = 14) and 16–17 years (43.8%, *n* = 14) groups. Reported growth arrest occurred in 25.4% of patients (*n* = 31), most commonly in the 13–15 years age group (42.4%, *n* = 14).

The mean tumor volume was 3.19 ± 5.76 cm³ (95% CI = 2.18–4.20), with no significant differences among the age groups (*p* = 0.4191) (Fig. 1e). In PPA/PitNETs, Knosp grading showed that 25.4% of tumors were grade 0, with grades 1, 2, 3, and 4 being less common and 50.8% of the tumors had no available Knosp grade data in the hospital charts. The upper intercarotid distance was 1.5 ± 0.4 cm (95% CI = 1.43–1.57, *p* = 0.4688), and the lower intercarotid distance was 2.0 ± 0.4 cm (95% CI = 1.93–2.07, *p* = 0. 0621), both showing no significant age-related difference. The mean width of the apertura piriformis was 1.6 ± 0.3 cm (95% CI = 1.55–1.65) with no significant differences across age groups (*p* = 0.0938).

The mean duration of surgery was 114.8 ± 58.5 min (95% CI = 1.55–1.65), with significant differences among age groups (*p* = 0.0009), being longest in the 3–9 years group (157.3 ± 76.7 min, 95% CI = 127.1–187.5.1.5) and shortest in the 16–17 years group (88.0 ± 37.9 min, 95% CI = 74.6–101.4.6.4) (Fig. 1f). EOR showed that 38.5% of patients had a total resection, 43.4% had a subtotal resection, and 14.8% had a biopsy across all entities, with no significant differences among the age groups (*p* = 0.6899). In addition, pneumatization positively correlated with age (R²=0.2721) with the youngest age group (3–9 years) showing a significantly decreased pneumatization (1.0 ± 0.8 cm; 95% CI = 0.71–1.29) (Fig. 1g). Moreover, pneumatization inversely correlated with operative duration, suggesting that more extensive sphenoid development facilitates surgical access and reduces operative time (Fig. 1h).

New or persistent postoperative deficits were observed in 53.3% of patients (*n* = 65), with a significantly higher incidence in the youngest age cohort (3–9 years) affecting 93.1% (*n* = 27/29) (*p* < 0.0001, OR = 19.5, 95% CI = 4.6–85.7) (Fig. 1i). In patients with preoperative visual impairment, persistent visual impairment was observed in 2.5% of all cases and no new postoperative visual impairments were reported in any case. New pituitary insufficiency occurred in 10.7% and tended to be more frequent in the youngest cohort (3–9 years: 6/29) than in older patients (10–17 years, 7/93) (OR = 3.21; 95% CI = 0.98–10.5). Permanent DI was noted in 24.6% of all patients, whereas transient DI appeared in 9.8% of all cases. The incidence of permanent DI was significantly higher in the 3–9 years age group (48.3%, *p* = 0.0045, OR = 4.49, 95% CI = 1.82–11.1), whereas transient DI was equally distributed in all age groups (*p* = 0.6619). In the overall cohort, there was a new onset of DI in 16.4% of all patients and in 18% patients initially presented with DI, with ongoing symptoms after surgery. Furthermore, other postoperative deficits included CSF leak (*n* = 4, 3.3%) and meningitis (*n* = 2, 3.5%). Further details will be presented by diagnosis.

Mean overall follow-up was 31.4 + 34.3 months. Recurrence or progression of the tumor was seen in 15.6% of patients, with the highest rate in the 3–9 years group (27.6%), though this was not statistically significant (*p* = 0.1118, OR = 2.84, 95% CI = 0.98–8.98). Tumor rest was observed in 36.8% of patients, with significant differences among age groups (*p* = 0.0363), being most common in the 3–9 years group (55.2%, OR = 2.72, 95% CI = 1.16–6.38). A total of 32 patients (26.2%) underwent adjuvant treatment, including re-surgery in 16 cases (13.1%). Radiotherapy alone (RTx) was administered to *n* = 9 patients (7.4%), while chemotherapy (CTx) was given in *n* = 1 patient (0.8%). Two patients received somatostatin analogs (SSA) (1.6%). No significant differences were observed between age groups regarding the need for adjuvant treatment (*p* = 0.1317).

We further examined the most common pediatric histological entities, focusing on craniopharyngioma and pituitary adenoma.

### Craniopharyngioma

**Fig. 2:**
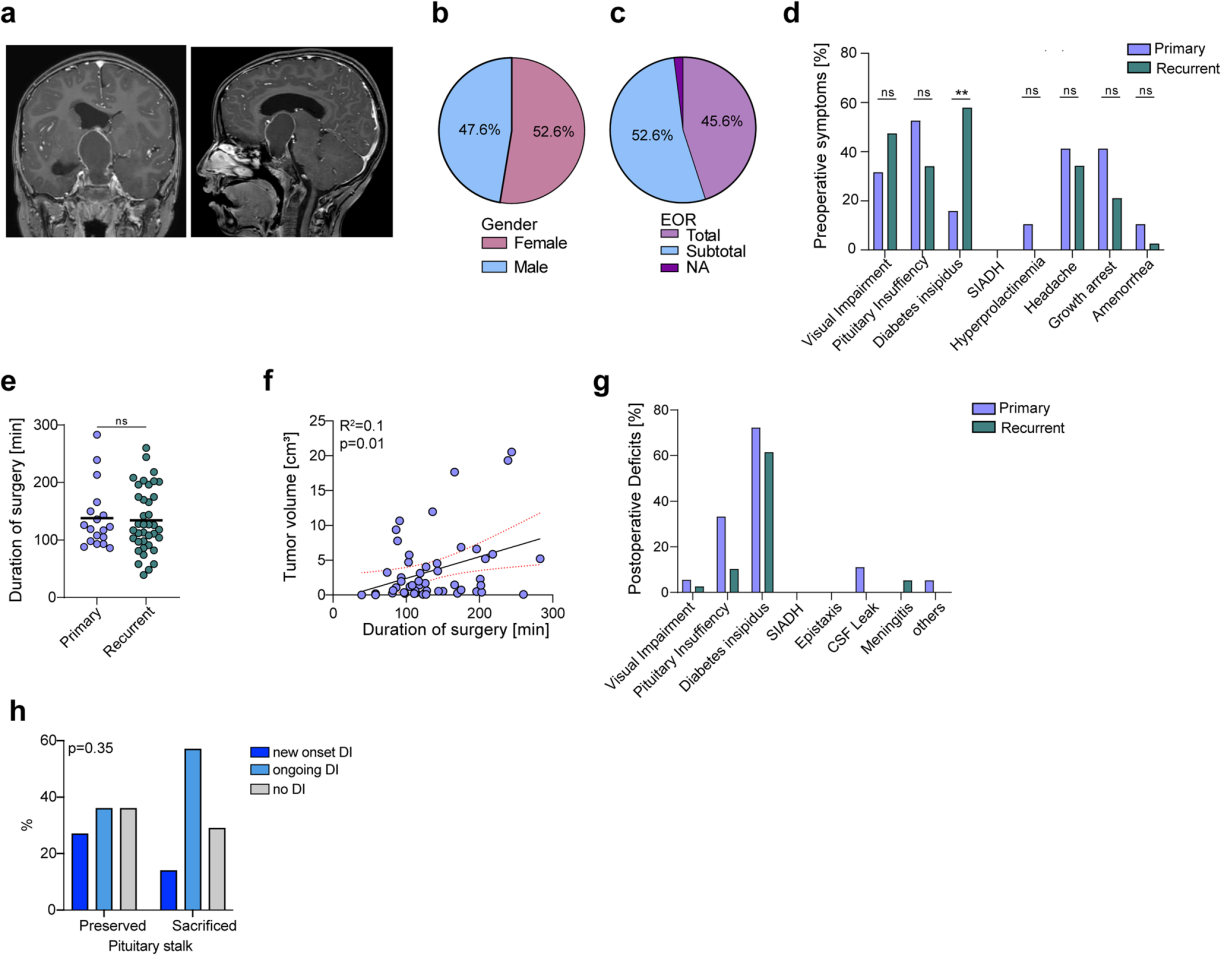
Craniopharyngioma **a** Coronal and sagittal T1-weighted MR-imaging of a craniopharyngioma. **b** Pie chart of sex-distribution in pediatric craniopharyngioma displays 52.6% female patients and 47.6% male patients. **c** In 45.6% of all patients a total resection could be achieved and in 52.6% a subtotal resection was performed. **d** Preoperative symptoms: The most frequent symptoms included visual impairment (42.1%), pituitary insufficiency (40.4%), and diabetes insipidus (DI) (43.9%). DI was notably more common in the recurrent group compared to the primary group (56.4% vs. 16.7%, *p* = 0.004). Additional symptoms were headaches (36.8%), growth arrest (28.1%), hyperprolactinemia (3.5%), and primary amenorrhea (3.5%). **e** Mean duration of surgery did not differ significantly from initial surgery to recurrent surgery (138 min. vs. 1134 min., *p* = 0.8654). **f** Linear regression shows no correlation between tumor volume and duration of surgery in minutes (R^2^ = 0.1220, *p* = 0.01). **g** After surgery, complications primarily manifested as new-onset pituitary insufficiency in first surgeries (33.3% vs. 10.3%) and permanent or transient DI (72.2% vs. 64.1%). **h** Correlation of pituitary stalk preservation and the occurrence of DI after surgery. New-onset DI occurred in 27.3% of patients with preserved and 14.3% with sacrificed stalks; ongoing DI in 36.4% and 57.1%, respectively; and no DI in 36.4% and 28.6% (*p* = 0.35)

Overall, craniopharyngioma patients represented the most common histological entity (*n* = 42, 34.4%) of surgically approached pediatric pituitary lesions and was most commonly found in younger age within the pediatric cohort (Fig. 2a). Characteristics of pediatric craniopharyngiomas and differences between primary and recurrent cases are summarized in Supplementary Table 1. Among a total of 42 individual patients with craniopharyngiomas, 57 transsphenoidal surgeries were performed, including 15 repeated procedures. Within the group of pediatric craniopharyngioma (Fig. 1a), a total of *n* = 18 (31.6%) presented with a primary tumor and *n* = 39 (68.4%) with a recurrent tumor.

Herein the overall median age was at 11 years (range: 4–17) and showed no significant differences between primary and recurrent craniopharyngeoma. In the overall cohort, both genders were equally distributed (Fig. 2b, Supplementary Table 1).

In *n* = 26 (45.6%) of all cases a total resection was achieved, whereas in *n* = 30 (52.6) of the patients a subtotal resection was performed (Fig. 2c). No biopsies were conducted, and one case in the recurrent group lacked EOR data. The most common preoperative symptoms were visual impairment (*n* = 22, 38.6%), pituitary insufficiency (*n* = 23, 40.4%), and DI (*n* = 25, 43.9%) (Fig. 2d). DI significantly more prevalent in the recurrent group compared to the primary group (56.4% vs. 16.7%, *p* = 0.004). Other preoperative symptoms included headaches (*n* = 21, 36.8%), growth arrest (*n* = 16, 28.1%), hyperprolactinemia (*n* = 2, 3.5%), and primary amenorrhea (*n* = 2, 2.5%), whereas secondary amenorrhea was not reported in craniopharyngioma (*n* = 0, 0%). The overall mean duration of surgery was 135.6 min and similar between primary and recurrent craniopharyngioma (138.1 ± 55.1 min vs. 134.3 ± 56.1 min, *p* = 0.8654) (Fig. 2e). Duration of surgery correlated with tumor volume (R^2^ = 0.1220, *p* = 0.01) (Fig. 2f). The most common postoperative complications were new-onset pituitary insufficiency (*n* = 13, 22.8%) and diabetes insipidus (DI) (*n* = 38, 66.7%) (Fig. 2g). Among these, 8.8% (*n* = 5) presented a transient DI, while 49.1% (*n* = 28) developed a permanent DI and 8.8% (*n* = 5) remained unknown. CSF leak occurred in 3.5% of all cases. There were no reported cases of postoperative SIADH, meningitis or epistaxis (Fig. 1g). Overall, recurrence occurred in *n* = 14 patients (24.6%). In addition, given the high rate of permanent postoperative DI, we examined whether preservation of the pituitary stalk reduced DI incidence. New-onset postoperative DI occurred at similar frequencies with stalk preservation versus sacrifice (27.3% vs. 14.3%; *p* = 0.35; Fig. 2h), indicating no significant association with neither intraoperative appearance nor imaging studies on functionality.

Residual tumor was present in 30 patients (52.6%), with *n* = 9 (47.4%) in the primary group and *n* = 21 (55.3%) in the recurrent group. In addition, after histopathological analysis, we observed that 98.2% of all pediatric craniopharyngioma showed an adamantinomatous subtype and we found only on case with a papillary subtype as a primary diagnosis at the age of 17 years, underscoring the rarity of papillary craniopharyngioma in children and its occurrence predominantly in late adolescence.

In our cohort (*n* = 57), BRAF V600E was tested in 21.1% (*n* = 12) and was negative in all tested cases; 78.9% (*n* = 45) had no BRAF result. CTNNB1 mutations were identified in 3/57 (5.2%). As adjuvant treatment, 6 patients (14.2%) received radiotherapy, 1 patient (2.4%) underwent radiation and re-surgery, and 14 patients (33.3%) underwent repeated surgery. The mean follow-up period was 38.56 ± 30.7 months overall.

### Pediatric pituitary adenoma (PPA)

**Fig. 3:**
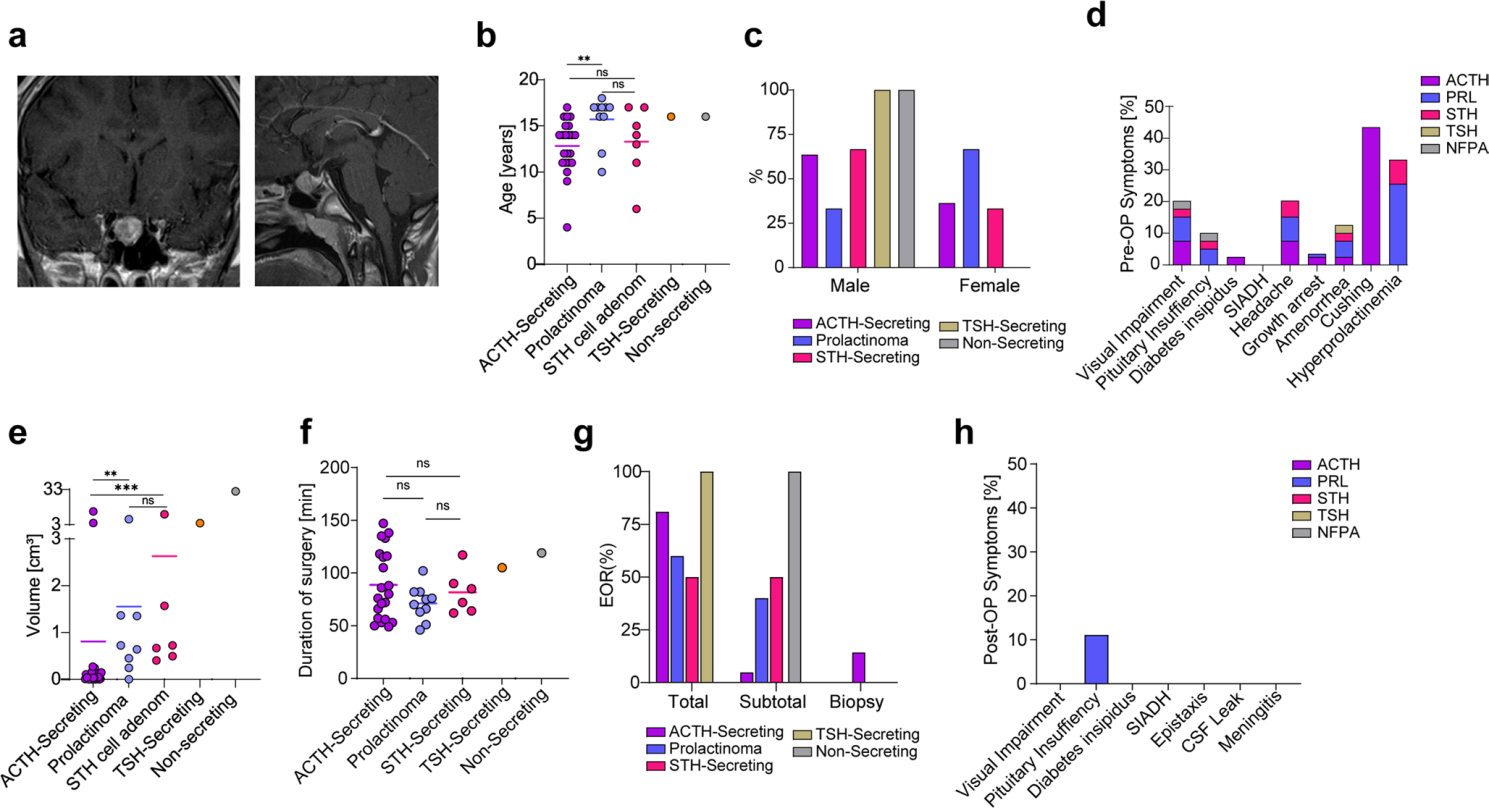
Pediatric pituitary adenoma **a** Coronal and sagittal T1-weighted MRI of a pediatric pituitary adenoma. **b** Age distribution of all PPA/PitNETs: ACTH-cell adenoma displayed a significantly younger age compared to PRL-cell adenoma (12 vs. 15.7, *p* = 0.0031). **c** Distribution of adenoma entities based on gender: There is a male predominance observed in ACTH- and STH-cell adenomas, while PRL-cell adenomas are more frequently found in females. **d** Distribution of preoperative symptoms within the different adenoma entities. **e** Adenoma volume was significantly smaller in ACTH-cell adenoma compared to PRL-cell adenoma (0.8 vs. 1.7 cm³, *p* = 0.0002) and to STH-cell adenoma (0.8 vs. 2.6, *p* = 0.0004). **f** Surgical duration did not significantly vary among different adenoma entities. **g** Extent of resection (EOR) was consistent across all adenoma entities. **h** Distribution of new postoperative symptoms following surgery

The second most common histological entity among surgically approached pediatric pituitary lesions were pituitary adenomas (*n* = 38, 31.5%), which were more frequently found in later childhood. Among the different PPA subtypes, ACTH-secreting adenoma were the most common (17.2%), followed by prolactinoma (7.4) and STH-secreting adenoma (4.9%) (Fig. 3a). Additionally, the mean age of ACTH-cell adenomas was significantly younger compared to patients with PRL-cell adenomas (12 vs. 15.7 years, *p* = 0.0031) (Fig. 3b). There was a gender difference observed among the different adenoma types, where ACTH-cell and STH-cell adenomas exhibited a male predominance, whereas PRL-cell adenomas were more frequently found in females (Fig. 3c). Preoperative symptoms included visual impairment and was equally distributed among the most common PPA (*p* = 0.2636) (Fig. 3d). Preoperative pituitary insufficiency was significantly higher in prolactinomas (20%) and non-secreting adenomas (100%) compared to ACTH-secreting adenomas (0%) (*p* = 0.0136). Preoperative DI was exclusively observed in ACTH-secreting adenomas (4.7%). In accordance with the diagnosis, hyperprolactinemia was seen in all prolactinomas (100%), but also concomitant in 33.3% of STH-secreting adenomas (*p* < 0.0001). Notably, only 5 of 9 patients with prolactinomas had been treated with dopamine agonists prior to surgery. Moreover, we observed that the volume of ACTH-cell adenomas was significantly smaller compared to both PRL-cell adenomas (0.8 cm³ vs. 1.7 cm³, *p* = 0.0002) and STH-cell adenomas (0.8 cm³ vs. 2.6 cm³, *p* = 0.0004) (Fig. 3e). Despite these differences in volume, the duration of surgery did not significantly vary among the different adenoma entities (Fig. 3f). Additionally, the EOR was consistent across all adenoma types, indicating uniform surgical outcomes (Fig. 3g).

Postoperative pituitary insufficiency was rare and only observed in one lactotrophic PitNET (*n* = 1/10, 10%) (Fig. 3h). No visual impairments or cases of DI, SIADH, CSF leakage, epistaxis, or meningitis were reported postoperatively in any of the adenoma subtypes. Persistently elevated hormone levels were observed in 4.8% (*n* = 1) of ACTH-secreting adenomas and in 33.3% (*n* = 3) of STH-secreting PitNETs. In contrast, all prolactinomas and TSH-secreting adenomas achieved complete normalization of hormone levels within three days post-surgery. Mean follow-up was 22.2 + 30.6 months. Recurrence rates were 4.8% for ACTH-secreting adenomas, 11.1% in prolactinoma and 16.6% for STH-secreting adenomas within a mean follow-up time of 14.6 + 27.4 months. The overall remission rate for all pediatric PitNETs was 84.2%. Re-surgery rates were 9.5% for ACTH-secreting adenomas, 11.1% for prolactinoma and 16.6% for STH-secreting adenomas.

To explore whether visualization modality influenced outcomes, we compared microscopic and exoscopic transsphenoidal techniques (Supplementary Table 2). Both techniques were feasible across all age groups with a median age of 12 in both groups. The exoscopic setup depicted a higher rate of GTR (47.9% vs. 36.0%, *p* = 0.0302) and a shorter duration of surgery (109 vs. 119 min, *p* = 0.015). Transient and permanent postoperative deficits were more frequent after microscopic surgery (17.3% vs. 5.6%, *p* = 0.037; 46.6% vs. 25.3%, *p* = 0.009). However, new permanent DI occurred more often in the exoscopic group (9.8% vs. 1.4%, *p* = 0.03).

## Discussion

In the current study, we present one of the largest and most recent series of transsphenoidal surgery in pediatric patients spanning a decade at a high-volume neurosurgical department in Germany. Besides the age-related differences in lesion subtypes with a wide variety of preoperative symptoms and postoperative complications, we can present a safe and effective surgical routine for a variety of pediatric sellar lesions even for younger children with no mortality.

A previous study from our institution demonstrated the safety and effectiveness of transsphenoidal surgery for pediatric patients with pituitary adenomas [10]. As an expansion, we incorporated these cases and adapted our series to a younger cutoff age of 18 years instead of 21 years, extended the study interval to 10 years, and investigated all histological entities addressed by transsphenoidal surgery in pediatric patients.

In this study, we demonstrated an age-dependent distribution pattern within all histological entities, highlighting the prominence of craniopharyngiomas in younger patients. In contrast, pituitary adenomas became increasingly prevalent in those older than 10 years, with corticotrophic and lactotrophic adenomas being the predominant subtypes. In a database study with an age cutoff of 21 years, PPA was identified as the most common diagnosis, followed by craniopharyngioma and germ cell tumors, with a higher prevalence in females. Craniopharyngeomas show a peak in early childhood, whereas PPA were more prevalent in males during middle childhood and their incidence increased in females around time of puberty [1, 3, 4, 10].

Moreover, our study investigated in age-specific anatomical considerations influencing transsphenoidal surgery. Herein, the variation in the size of sphenoid pneumatization implies possible intraoperative challenges for surgical approaches of the sellar region. Previous studies have shown that sphenoid sinus pneumatization is absent in children under 3 years old but is fully present by ages 8–11, as observed in a series of 60 pediatric patients [8, 9]. In our series, the youngest patient was three years old, and we observed a clear correlation between pneumatization and patient age across the entire cohort. In addition, Oveido et al. described a reduced risk of intraoperative bleeding with greater pneumatization of the sphenoid sinus and decreased pneumatization being associated with worse visualization of anatomical landmarks that increase the risk of vascular injury [8]. In our series, we did not observe a higher rate of complications in younger patients, but we did find a significantly longer surgery duration in the youngest age group (3–9 years), reflecting these anatomical challenges. Surgery duration did not significantly differ among the other, older age groups, aligning with the progression of skull base maturation. Sellar development shows partly linear growth in early to mid-childhood with significant growth in late adolescence (16–18 years), indicating anatomical stability well after puberty [9]. Additionally, small nasal and piriform apertures and a decreased inter-carotid interval of around 8–10 mm are age-related factors surgeons should consider. However, in our series, the mean upper and lower intercarotid distance was above 1 cm across all age groups and we did not identify any significant differences in perioperative outcomes related to these anatomical considerations.

Our findings reinforce that craniopharyngioma and PitNETs require distinct surgical strategies and outcome reporting, and that pooling these entities as “pituitary tumors” may mask clinically relevant differences in risk profiles and postoperative endocrine sequelae.

In craniopharyngioma, its pediatric management remains challenging and should be administered in a multidisciplinary setting, especially when highlighting the rate of perioperative complications with subsequent morbidity and the high rate for adjuvant treatment. Our cohort represents these interdisciplinary challenges indirectly by the high rate of subtotal resection in the primary setting and the high rate of secondary surgeries carried out in our center. Additionally, a wide range of preoperative and postoperative complications, particularly DI and pituitary insufficiency, which carry high morbidity, place significant emphasis on perioperative care. Notably, in primary addressed craniopharyngeomas the rate of postoperative DI in our cohort was as high as 70% following the initial surgical intervention, even with a high rate of subtotal resections, aligning with previous studies [11]. DI was especially persistent and significantly more prevalent during recurrent surgeries in our cohort.

The need for adjuvant treatment during a 2-3years follow up period is about 20%, and recurrence occurred in 26.2% of cases in our series. A recent retrospective analysis of 80 pediatric patients confirmed this by a rate of 37,5% for recurrence after primary surgery within a mean follow up of 10 years and with subtotal resection without adjuvant therapy being an independent risk factor [12]. Gross total resection (GTR) has been accomplished in about 50% of cases in this retrospective monocenter study in comparison to 45% in our study with only transsphenoidal approaches. Additionally, prior studies stated that EOR is the most important factor for long-term disease control with a 10-years recurrence rate of 0–50% after GTR and 25–100% after STR [11]. However, pan hypopituitarism is a common postoperative complication, especially linked to GTR [12] and is present in about 50 to almost 100% postoperatively [11]. Therefore, balancing the risk and benefits for aggressive surgical strategies should be considered. GTR should be reviewed critically.

In our study, pituitary adenomas (PPAs) were the second most common histological entity (31.5%) operated on in children using the transsphenoidal approach. We observed that corticotrophic adenomas were more prevalent in younger children, while the incidence of lactotrophic adenomas increased during adolescence [13]. According to the latest Endocrine Society guidelines, it is expected that a growing number of microprolactinomas, including those in younger patients, will be managed primarily through surgical intervention. Consistent with results from other studies [2, 7, 8], ACTH-secreting adenomas accounted for more than half of PPAs, whereas non-secreting adenomas represented only about 4% in childhood. Additionally, there is an age-dependent pattern: ACTH-secreting adenomas predominate before puberty, while prolactinomas and GH-secreting adenomas become more common after puberty [1, 4]. A relationship between tumor size and hormone secretion has also been evaluated for the latter [1].

Interestingly, ACTH-secreting adenomas require similar operating times despite being smaller in size and volume than other entities. This may be due to the difficulty in intraoperative distinction and the special care needed for total resection, as surgical cure is critical for this entity, which has the highest long-term recurrence rate among secreting adenomas [14]. As ACTH-secreting adenoma are more prevalent in younger children, the smaller surgical corridor due to age-related anatomical variations in sphenoid sinus pneumatization, may also influence operating time for ACTH-secreting adenomas.

In the long term, recurrent or persistent disease has been reported in about 54% and 35% of cases, respectively, with a mean follow-up of 63 months in a literature review of 37 publications after primary surgery [14]. Compared to adult pituitary adenomas, a significant fraction of pediatric cases presented with recurrent/persistent disease with a surgical cure rate of around 50–65% [14]. However, in our cohort, this fraction is smaller than in previous studies, where recurrent surgery remains infrequent in our cohort, with a remission rate of 82.4% within a mean follow-up interval of 14.6 months.

Therefore, PPAs appear more prone to recurrence in children and may pose a challenge for multimodal treatment algorithms, as recurrent surgery may lead to a cure in only about 30% of pediatric cases, compared to up to 60% in the adult population. Moreover, disease- or treatment-associated hypopituitarism can have dramatic implications in children, such as growth arrest and infertility [14]. Decision-making in cases of recurrence is highly individualized based on patient characteristics, age, and lesion type.

Most current literature describe safety and efficacy of endoscopy sellar surgery with mixed results in terms of outcome variables and surgical morbidities when comparing to microscopic technique. Comparable results has been shown for both techniques in infradiaphramtic lesions with a rate of GTR of around 66% in a recent review [15]. Recurrence rate was higher in endoscopic series, but mortality or overall complication rate were similar with a rate of CSF leakage of 13% [15].

When comparing our results with the literature, most recent pediatric series report comparable or even higher rates of gross total resection after endoscopic transsphenoidal approaches, but often at the cost of increased postoperative morbidity, particularly regarding endocrine dysfunction and cerebrospinal fluid (CSF) leakage [16–18]. In our cohort, despite a high proportion of recurrent tumors and the absence of fully endoscopic visualization, we achieved favorable surgical and functional outcomes within the expected range of published pediatric series. These findings emphasize that both microscopic and exoscopic transsphenoidal techniques offer excellent visualization and surgical control while maintaining low complication rates, supporting their continued use in carefully selected pediatric sellar and parasellar lesions.

### Limitations

This study is a retrospective, single-center investigation. Since only neurosurgically treated patients were included, selection bias is possible, and the results may not apply to conservatively managed sellar lesions. A key limitation to consider is that comparisons between studies and case series are complicated by differing age classifications and cutoff values used to define the pediatric population, which range from 16 to 21 years [1, 10]. Notably, some studies exclude the youngest age group, particularly children under 12 years old [6, 14] This underscores the importance of our study, which includes very young patients, even those below 12 years, who underwent surgical therapy for specific indications. As this series exclusively includes transsphenoidal cases, no direct comparison with transcranial approaches could be performed. Therefore, the results mainly reflect the safety profile and outcomes of transsphenoidal techniques in anatomically suitable cases. Molecular profiling (BRAF V600E, CTNNB1) was not routinely performed during the early years of the study. Given the substantial proportion of untested cases, these results should be interpreted with caution. Further comparison surgery-associated morbidities of microscopic and endoscopic technique is limited due to the necessity to compare the small cohort size for different entities.

Although our study includes a relatively large cohort, it is important to concede that pediatric sellar lesions requiring transsphenoidal surgery are rare, and the available evidence remains limited due to the typically small sample sizes in this population.

## Conclusion

This study is one of the largest to date, analyzing perioperative features of pediatric pituitary region lesions in 122 patients surgically treated between 2013 and 2023. The age-dependent distribution pattern reveals the prominence of craniopharyngiomas in younger patients, while PPAs became more common in those older than 10 years, with corticotrophic and lactotrophic adenomas being the most prevalent subtypes. In addition, younger patients tend to have longer surgery durations due to less pneumatized sphenoid sinuses, indicating that anatomical maturity facilitates easier surgical access.

Craniopharyngiomas, the most frequent subtype, present with a wide range of pre- and postoperative complications, a high rate of subtotal resections, and a significant need for adjuvant multimodal treatment. This necessitates a specialized approach to manage the high morbidity associated with these lesions and their treatments. In contrast, PPAs can typically be treated safely with transsphenoidal microsurgical resection, even in younger children, without a significantly higher complication rate and good remissions rates of more than 80% when treated in specialized centers.

## Supplementary Information

Below is the link to the electronic supplementary material.

Supplementary Material 1

Supplementary Material 2

## Data Availability

No datasets were generated or analysed during the current study.

## Abbreviations

ACTH: Adrenocorticotropic hormone
CSF: Cerebro Spinal Fluid
DI: Diabetes insipidus
EOR: Extent of resection
GH: Growth hormone
GTR: Gross total resection
MRI: Magnetic resonance imaging
PitNET: Pituitary neuroendocrine tumor
PPA: Pediatric pituitary adenoma
PRL: Prolactin
SIADH: Syndrome of inappropriate antidiuretic hormone secretion
STH: Somatotropic hormone
STR: Subtotal resection
