# Supplementary material for: Contemporary series of transsphenoidal microsurgery in pediatric patients
Source: Neurosurg Rev. 2026 Jan 23;49(1):144. doi: 10.1007/s10143-025-04019-6 (PMC12827427; doi:10.1007/s10143-025-04019-6)
Supplement: Supplementary file 1 — Supplementary Material 1 [file 10143_2025_4019_MOESM1_ESM.docx]

| **Supplementary Table 1** | | |  |  |  |  |
| --- | --- | --- | --- | --- | --- | --- |
|  |  |  | **All** | **Primary** | **Recurrent** | p-value |
| Total - n (%) | | | 57 | 18 (31.6) | 39 (68.4) |  |
|  |  |  |  |  |  |  |
| Clinical | |  |  |  |  |  |
|  | Gender - n (%) | |  |  |  | 0.409 |
|  |  | Female | 30 (52.6) | 11 /18 (61.1) | 19 (48.7) |  |
|  |  | Male | 27 (47.4) | 7/18 (38.9) | 20 (51.3) |  |
|  | Age, median (range) [yrs] | | 11 (4-17) | 11 (4-17) | 11 (4-17) | 0.726 |
|  | Preoperative symptoms | |  |  |  |  |
|  |  | Visual Impairment - n (%) | 22 (38.6) | 6/18 (30) | 16/39 (41) | 0.393 |
|  |  | Pituitary Insufficiency - n (%) | 23 (40.4) | 10/18 (55.6) | 13/39 (33.3) | 0.253 |
|  |  | Diabetes insipidus - n (%) | 25 (43.9) | 3/18 (16.7) | 22/39 (56.4) | **0.004** |
|  |  | SIADH - n (%) | 0 (0) | 0/18 (0) | 0/39 (0) | >0.999 |
|  |  | Hyperprolactinemia - n (%) | 2 (3.5) | 2/18 (11.1) | 0/39 (0) | 0.107 |
|  |  | Headache - n (%) | 21 (36.8) | 8/18 (44.4) | 13/39 (33.3) | 0.574 |
|  |  | Growth arrest - n (%) | 16 (28.1) | 8/18 (44.4) | 8/39 (20.5) | 0.123 |
|  |  | Amenorrhoe - n (%) | 3 (5.3%) | 2/18 (11.1) | 1/39 (2.6) | 0.255 |
|  |  | Primary | 2 (3.5) | 1/18 (5.5) | 1/39 (2.6) |  |
|  |  | Secondary | 0 (0) | 0/18 (0) | 0/39 (0) |  |
|  |  | NA | 1 (1.7) | 1/18 (5.5) | 0/39 (0) |  |
| Radiographic parameters | | |  |  |  |  |
|  |  | Volume [cm3] - mean + SD | 3.6 + 4.8 | 5.1 + 6.1 | 2.9 + 4.1 | 0.076 |
|  |  | Cystic - n (%) | 16 (28.1) | 6/18 (30) | 10/39 (25.6) | 0.148 |
|  |  | Hydrocephalus - n (%) |  |  |  | >0.999 |
|  |  | present | 4 (7) | 2/18 (11.1) | 2/39 (5.1) |  |
|  |  | absent | 17 (29.8) | 8/18 (44.4) | 9/39 (23.1) |  |
|  |  | NA | 36 (63.2) | 8/18 (44.4) | 28/39 (71.8) |  |
|  |  | Compressing chiasm - n (%) |  |  |  | 0.361 |
|  |  | present | 14 (24.6) | 8/18 (44.4) | 6/39 (15.4) |  |
|  |  | absent | 7 (12.3) | 2/18 (11.1) | 5/39 (12.8) |  |
|  |  | NA | 36 (63.2) | 8/18 (44.4) | 28/39 (71.8) |  |
|  |  | Puget classification - n (%) |  |  |  | 0.387 |
|  |  | 0 | 13 (22.8) | 5/18 (27.8) | 8/39 (20.5) |  |
|  |  | 1 | 8 (14) | 5/18 (27.8) | 3/39 (7.8) |  |
|  |  | 2 | 0 (0) | 0/18 (0) | 0/39 (0) |  |
|  |  | NA | 36 (63.2) | 8/18 (44.4) | 28/39 (71.8) |  |
|  |  | Kassam classification - n (%) |  |  |  | 0.629 |
|  |  | Type I | 6 (10.5) | 2/18 (11.1) | 4/39 (10.3) |  |
|  |  | Type II | 4 (7) | 3/18 (16.7) | 1/39 (2.6) |  |
|  |  | Type IIIa | 9 (15.8) | 4/18 (22.2) | 5/39 (12.8) |  |
|  |  | Type IIIb | 2 (3.5) | 1/18 (5.5) | 1/39 (2.6) |  |
|  |  | Type IV | 0 (0) | 0/18 (0) | 0/39 (0) |  |
|  |  | NA | 36 (63.2) | 8/18 (44.4) | 28/39 (71.8) |  |
| Histological parameters | | |  |  |  | 0.316 |
|  |  | Adamantinous - n (%) | 56 (98.2) | 17/18 (94.4) | 39/39 (100) |  |
|  |  | Papillary - n (%) | 1 (1.8) | 1/18 (5.5) | 0/39 (0) |  |
|  |  | BRAF mutation - n (%) |  |  |  |  |
|  |  | Mutated | 0 (0) | 0/18 (0) | 0/39 (0) |  |
|  |  | Not mutated | 12 (21.1) | 3/18 (16.7) | 9/39 (23.1) |  |
|  |  | NA | 45 (78.9) | 15/18 (83.3) | 30/39 (76.9) |  |
|  |  | CTNNB1 Mutation - n (%) | 3 (5.2) | 1/18 (5.5) | 2/39 (5.1) |  |
| Surgical parameters | | |  |  |  |  |
|  | EOR | |  |  |  | >0.999 |
|  |  | Total | 26 (45.6) | 8/18 (44.4) | 18/39 (46.2) |  |
|  |  | Subtotal | 30 (52.6) | 10/18 (55.6) | 20/39 (51.3) |  |
|  |  | Biopsy | 0 (0) | 0/18 (0) | 0/39 (0) |  |
|  |  | NA | 1 (1.8) | 0/18 (0) | 1/39 (2.6) |  |
|  | Duration of surgery [min] - Mean + SD | |  | 138.1 + 55.1 | 134.3 + 56.1 | 0.865 |
| Outcome | |  |  |  |  |  |
|  |  | Pituitary stalk - n (%) |  |  |  | 0.162 |
|  |  | preserved | 22 (38.6) | 8/18 (44.4) | 14/39 (35.9) |  |
|  |  | sacrified | 21 (36.8) | 3/18 (16.7) | 18/39 (46.2) |  |
|  |  | NA | 14 (24.6) | 7/18 (38.9) | 7/39 (17.9) |  |
|  | Postoperative Deficits (Persistent/New) | |  |  |  |  |
|  |  | Persistent Visual Impairment - n (%) | 2 (3.5) | 1/18 (5.5) | 1/39 (2.6) |  |
|  |  | New Pituitary Insufficiency - n (%) | 10 (17.5) | 6/18 (33.3) | 4/39 (10.3) | 0.081 |
|  |  | Diabetes insipidus - n (%) | 37 (64.9) | 13 /18 (72.2) | 24/39 (61.5) | 0.555 |
|  |  | Persistent | 24 (42.1) | 3/18 (16.7) | 21/39 (53.8) |  |
|  |  | New onset | 13 (22.8) | 10/18 (55.6) | 3/39 (7.7) |  |
|  |  | transient | 5 (8.8) | 3/18 (16.7) | 2/39 (5.1) |  |
|  |  | permanent | 27 (47.4) | 9/18 (50) | 18/39 (46.2) |  |
|  |  | NA | 5 (8.7) | 1/18 (5.5) | 4/39 (10.3) |  |
|  |  | SIADH - n (%) | 0 (0) | 0/18 (0) | 0/39 (0) | >0.999 |
|  |  | Epistaxis - n (%) | 0 (0) | 0/18 (0) | 0/39 (0) | >0.999 |
|  |  | CSF Leak - n (%) | 2 (3.5) | 2/18 (11.1) | 0/39 (0) | 0.095 |
|  |  | Meningitis - n (%) | 0 (0) | 0/18 (0) | 2/39 (5.1) |  |
|  | Recurrence - n (%) | | 14 (24.6) | 3/18 (16.7) | 11/39 (28.2) | 0.511 |
|  | FUP, mean (SD) [months] | | 38.56 + 30.7 | 34.86 + 29.59 | 40.42 + 31.71 |  |
|  |  |  |  |  |  |  |
