# Supplementary material for: Contemporary series of transsphenoidal microsurgery in pediatric patients
Source: Neurosurg Rev. 2026 Jan 23;49(1):144. doi: 10.1007/s10143-025-04019-6 (PMC12827427; doi:10.1007/s10143-025-04019-6)
Supplement: Supplementary file 2 — Supplementary Material 2 [file 10143_2025_4019_MOESM2_ESM.docx]

| **Supplementary Table 2** | |  |  |  |
| --- | --- | --- | --- | --- |
|  |  | **Microscopic** | **Exoscopic** | p-value |
| Total - n (%) | | 75 | 71 |  |
| Age - median (range) | | 12 (4-17) | 12 (3-17) |  |
| EOR |  |  |  | 0.057 |
|  | Total | 27/75 (36) | 34/71 (47.9) | **0.030** |
|  | Subtotal | 40/75 (53.3) | 22/71 (31) |  |
|  | Biopsy | 7/75 (9.3) | 9/71 (12.7) |  |
|  | NA | 1/75 (1.4) | 6/71 (8.4) |  |
| Duration of surgery [min] - Mean + SD | | 119.5 + 47.7 | 109 + 64.1 | **0.015** |
| **Outcome** | |  |  |  |
| Postoperative Deficits | | 48/75 (64) | 22/71 (30.9) | **<0.0001** |
| Transient | | 13/75 (17.3) | 4/71 (5.6) | **0.037** |
| Diabetes insipidus - n (%) | |  |  |  |
| Persistent after surgery | | 0/75 (0) | 0/71 (0) |  |
| New onset | | 8/75 (10.7) | 4/71 (5.6) | 0.369 |
| SIADH - n (%) | | 0/75 (0) | 0/71 (0) |  |
| CSF Leak - n (%) | | 2/75 (2.7) | 0/71 (0) | 0.497 |
| Meningitis - n (%) | | 2/75 (2.7) | 0/71 (0) | 0.497 |
| Others - n (%) | | 1/75 (1.4) | 0/71 (0) | >0.999 |
| Permanent | | 35/75 (46.6) | 18/71 (25.3) | **0.009** |
| Visual Impairment - n (%) | | 3/75 (4) | 0/71 (0) | 0.245 |
| New Pituitary Insufficiency - n (%) | | 8/75 (10.7) | 6/71 (8.4) | 0.781 |
| Diabetes insipidus - n (%) | |  |  |  |
| Persistent after surgery | | 23/75 (30.7) | 5/71 (7) | **0.003** |
| New onset | | 1/75 (1.4) | 7/71 (9.8) | **0.030** |
| SIADH - n (%) | | 0/75 (0) | 0/71 (0) |  |
| Recurrence - n (%) | | 17/75 (22.7) | 3/71 (42.3) | **0.0013** |
